# The plasma membrane–associated Ca^2+^ ‐binding protein, PCaP1, is required for oligogalacturonide and flagellin‐induced priming and immunity

**DOI:** 10.1111/pce.14118

**Published:** 2021-06-30

**Authors:** Moira Giovannoni, Lucia Marti, Simone Ferrari, Natsuki Tanaka‐Takada, Masayoshi Maeshima, Thomas Ott, Giulia De Lorenzo, Benedetta Mattei

**Affiliations:** ^1^ Department of Biology and Biotechnology “C. Darwin” Sapienza University of Rome Rome Italy; ^2^ Laboratory of Cell Dynamics, Graduate School of Bioagricultural Sciences Nagoya University Nagoya Japan; ^3^ Faculty of Biology, Cell Biology University of Freiburg Freiburg Germany; ^4^ CIBSS ‐ Centre for Integrative Biological Signalling Studies University of Freiburg Freiburg Germany; ^5^ Department of Health, Life and Environmental Sciences University of L'Aquila L'Aquila Italy

**Keywords:** *Arabidopsis thaliana*, *Botrytis cinerea*, elicitor‐induced resistance, flagellin, microdomains, oligogalacturonides, plant immunity

## Abstract

Early signalling events in response to elicitation include reversible protein phosphorylation and re‐localization of plasma membrane (PM) proteins. Oligogalacturonides (OGs) are a class of damage‐associated molecular patterns (DAMPs) that act as endogenous signals to activate the plant immune response. Previous data on early phosphoproteome changes in *Arabidopsis thaliana* upon OG perception uncovered the immune‐related phospho‐regulation of several membrane proteins, among which PCaP1, a PM‐anchored protein with actin filament–severing activity, was chosen for its potential involvement in OG‐ and flagellin‐triggered responses. Here, we demonstrate that PCaP1 is required for late, but not early, responses induced by OGs and flagellin. Moreover, *pcap1* mutants, unlike the wild type, are impaired in the recovery of full responsiveness to a second treatment with OGs performed 24 h after the first one. Localization studies on PCaP1 upon OG treatment in plants expressing a functional PCaP1‐GFP fusion under the control of *PCaP1* promoter revealed fluorescence on the PM, organized in densely packed punctate structures, previously reported as microdomains. Fluorescence was found to be associated also with endocytic vesicles, the number of which rapidly increased after OG treatment, suggesting both an endocytic turnover of PCaP1 for maintaining its homeostasis at the PM and an OG‐induced endocytosis.

## Abstract 2

The *A. thaliana* plasma membrane (PM)‐anchored protein, PCaP1, is involved in elicitor‐induced protection against the necrotrophic fungus *B. cinerea*.

(GFP)‐PCaP1 organization into PM microdomains and its oligogalacturonide‐induced endocytosis point to a role of PCaP1 and its turn‐over in the sensing/transduction response to elicitors.

## 1 INTRODUCTION

Plants evolved various mechanisms to counteract pathogen attacks. Some of these provide constitutive physical and chemical barriers to pathogen infections, while others are induced only upon pathogen perception (Boller & Felix, 2009). An innate immune system is crucial for plant survival and is characterized by a rapid activation of defense responses triggered by the perception of danger signals (Chisholm et al., 2006) mediated by specific pattern recognition receptors (PRRs) (Albert et al., 2020; Boutrot & Zipfel, 2017). Among the danger signals, pathogen‐/microbe‐associated molecular patterns (PAMPs/MAMPs) are conserved molecules secreted or present on the surface of microbial pathogens that are capable of activating the so‐called pattern‐triggered immunity (PTI) against a wide range of pathogens. Plants are also capable of activating the immune system by sensing endogenous molecular patterns present only when the tissue is infected or damaged (damage‐associated molecular patterns or DAMPs), thus discriminating between an intact and an altered self (Duran‐Flores & Heil, 2018; Hou et al., 2019).

Oligogalacturonides (OGs) are a well‐known class of DAMPs (De Lorenzo et al., 2018; De Lorenzo et al., 2019; Ferrari et al., 2013; Pontiggia et al., 2020). Upon infection, phytopathogenic microbes degrade homogalacturonan (HGA), the main component of pectin, by utilizing *endo*‐polygalacturonases (PGs) and other pectic enzymes. In the cell wall, the interaction between PGs and polygalacturonase‐inhibiting proteins (PGIPs) enhances the formation of OGs (Benedetti et al., 2015; D'Ovidio et al., 2004; Mattei et al., 2005) capable of inducing a variety of plant defences (Ferrari et al., 2013; Galletti et al., 2011). Treatment with OGs protects *Arabidopsis thaliana*, grapevine (*Vitis vinifera*) and tomato (*Solanum lycopersicum*) against infections by the necrotrophic fungus *Botrytis cinerea (*Aziz et al., 2007 *;* Ferrari et al., 2007; Gamir et al., 2020 *)*. OGs not only act as DAMPs but also as negative regulators of plant growth and development mainly through their antagonism with auxin (Bellincampi et al., 1993; Ferrari et al., 2013; Pontiggia et al., 2020; Savatin et al., 2011).

The surface receptor kinase wall‐associated kinase 1 (WAK1) has been shown to mediate perception of OGs (Brutus et al., 2010; Gramegna et al., 2016; Vaahtera et al., 2019). WAKs are considered sensors (De Lorenzo et al., 2011; Kohorn, 2016) of the cell wall integrity (CWI) and part of the system that perceives CW alterations and coordinates the restoration of the CW functional integrity and cell growth (Engelsdorf et al., 2018; Vaahtera et al., 2019).

As shown by transcriptome analysis, early responses induced by OGs largely overlap with those induced by flg22, a MAMP derived from the bacterial flagellin. The production of reactive oxygen species (ROS), changes in ion fluxes and deposition of callose are also common responses to OGs and MAMPs (Ferrari et al., 2013; Gravino et al., 2017).

Most of the mechanisms by which the OG signal is transduced are not yet known. Protein phosphorylation/de‐phosphorylation and other post‐translational modifications (PTMs) are likely to play a role in the response to OGs (Macho & Zipfel, 2014; Withers & Dong, 2017). Indeed, kinases such as calcium‐dependent protein kinases (CDPKs) (Bigeard et al., 2015; Gravino et al., 2015) and mitogen‐activated protein kinases (MAPKs) are important components of the OG and MAMP‐induced immune response. For example, in Arabidopsis, the MAPKs indicated as MPK3 and MPK6 (Asai et al., 2002; Galletti et al., 2011) and the MAP kinase kinase kinases ANPs (Arabidopsis NPK1‐related protein kinases) (Marti et al., 2020; Savatin et al., 2014) play a major role in the response to OGs and flg22.

A large‐scale study of early phosphoproteome changes in Arabidopsis upon OG perception allowed to uncover the phospho‐regulation of more than 90 membrane proteins and suggested that an interplay occurs between several processes such as intracellular trafficking and vesicle dynamics, cytoskeleton rearrangement, signal transduction and phospholipid signalling (Mattei et al., 2016). In particular, among the OG‐dependent phosphorylated proteins, we found the plasma membrane cation binding protein 1 (PCaP1), also known as microtubule‐destabilizing protein 25 (MDP25). PCaP1 is a hydrophilic protein belonging to the plant‐specific DREPP (developmentally regulated plasma membrane polypeptide) family, the members of which are characterized by a peripheral interaction with the PM and differential regulation during plant development (Gantet et al., 1996; Logan et al., 1997; Vosolsobe et al., 2017). PCaP1 is anchored to the PM through both N‐myristoylation at glycine 2 (Gly 2) and a relatively strong polybasic amino acid cluster in the N‐terminal region that has been shown to bind phosphatidylinositol phosphates (PIPs) in vitro experiments (Nagasaki et al., 2008; Vosolsobe et al., 2017). In particular, interaction has been observed with phosphatidylinositol 4,5‐bisphosphate [PI(4,5)P_2_]_,_ a biochemical landmark of PM, and more strongly with PI(3,5)P_2_, which however is less abundant in plant cells and localized in late endosomes and tonoplast (Gerth et al., 2017; Xing et al., 2020). These observations suggest that the localization of PCaP1 might be regulated by the presence of the lipids on the PM, vesicles or endomembranes.

PCaP1 also interacts with Ca^2+^ and with Ca^2+^‐calmodulin (CaM) complexes in a Ca^2+^‐dependent manner (Kato et al., 2010; Li et al., 2011; Nagasaki et al., 2008). CaM weakens the interaction with PIPs but does not interfere with PCaP1 membrane localization (Kato et al., 2010). Very high, non‐physiological Ca^2+^ levels have been shown to induce the dissociation of PCaP1 from the PM, its release into the cytosol and its binding to filaments of the cortical cytoskeleton, leading to their destabilization (Li et al., 2011). More recently, PCaP1 has been shown to be an actin‐binding protein (ABP) that interacts directly with actin, through the 23‐amino acid N‐terminal region that also binds PIPs (Vosolsobe et al., 2017), and to sever individual actin filaments (Qin et al., 2014). The action on the cytoskeleton is likely responsible for the capability of PCaP1 to negatively regulate hypocotyl elongation (Li et al., 2011), pollen tube growth (Qin et al., 2014) and the root hydrotropic response (Tanaka‐Takada et al., 2019).

The observations that *PCaP1* expression is induced following treatment with Cu^2+^ (Nagata et al., 2016) and flg22 (Ide et al., 2007) and that PCaP1 is phosphorylated in response to flg22 (Rayapuram et al., 2014) and OGs suggest a role in immunity. In this work, we demonstrate that PCaP1 plays a role in PTI and is required for a full response to OGs. Moreover, we show that the protein is organized in PM microdomains and is internalized in endocytic vesicles in response to OGs.

## 2 MATERIAL AND METHODS

### 2.1 Plant materials

Wild‐type seeds of *Arabidopsis thaliana* ecotype Columbia‐0 (Col‐0) were purchased from Lehle Seeds *(Round Rock, TX, USA)*. Seeds of the T‐DNA insertional mutant *pcap1‐1* (SALK_022955 line) *and pcap1‐3 (GABI_872_G04) were obtained from The Nottingham Arabidopsis Stock Centre (NASC)* (School of Biosciences, University of Nottingham, United Kingdom). Homozygous mutants were isolated by PCR‐based genotyping using the gene‐specific PCR primers listed in Table S1 and primers for the T‐DNA sequence (Lba1 for Arabidopsis SALK mutant line and 8474 for Arabidopsis GABI Kat mutant line). Transgenic proPCaP1:PCaP1‐GFP seedlings were generated in a previous work (Nagata et al., 2016). Complemented lines proPCaP1‐GFP/*pcap1‐1* and proPCaP1‐GFP/*pcap1‐3* were obtained crossing the proPCaP1:PCaP1‐GFP line with *pcap1‐1* and *pcap1‐3* null mutants. Double‐homozygous were confirmed by PCR‐based genotyping and confocal microscopy for the fusion protein. F4 progeny was used for complementation assay.

### 2.2 Growth conditions and plant treatments

Arabidopsis plants were grown on soil (Compo Sana) at 22°C, 70% relative humidity under 12/12 h light/dark cycle *(*approximately 120 μmol m^−2^ s^−1^). For seedling assays, seeds were surface sterilized *using a solution composed of 0.01% w/v sodium dodecyl sulfate, 1.6% v/v NaClO for 10* min*, washed* and germinated in multi‐well plates (approximately 10 seeds well^−1^) containing 0.5X MS (Murashige and Skoog, 1962) medium supplemented with 0.5% sucrose (2 ml well^−1^).

For gene expression and immunoblotting analysis, seedlings were grown at 22°C and 70% relative humidity under a 12/12 h light/dark cycle (approximately 120 μmol m^−2^ s^−1^). After 9 days, the medium was adjusted to 1 ml, and treatments with OGs (50 μg ml^−1^) and flg22 (10 nM) were performed after 24 h.

For desensitization in seedlings, after 9 days, the medium was adjusted to 1 ml, and pre‐treatments with OGs (50 μg ml^−1^) and water, as a control, were performed. 24 h later, seedlings were treated again with OGs (50 μg ml^−1^) and water.

For the protection assay, 4‐week‐old plants were sprayed with water, OGs (200 μg ml^−1^) and flg22 (1 μM). For ROS production analysis, leaf discs were obtained from 4‐week‐old plants.

OGs with an average DP of 10 to 15 were obtained as previously described (Benedetti et al., 2017; Pontiggia et al., 2015).

### 2.3 Genomic DNA extraction

Leaves were frozen in liquid nitrogen and homogenized with a MM301 Ball Mill (Retsch, Basel, Switzerland) for about 1 minute at 24 Hz. Homogenate was solubilized with 200 μL of extraction buffer (200 mM Tris–HCl pH 7.5, 25 mM EDTA,150 mM NaCl, 0.5% v/v SDS) and 5 μL of RNase (10 mg ml^−1^) (Ribonuclease A, Sigma‐Aldrich®) and mixed. The mixture was incubated at 56°C for 30 min and then centrifuged at 13 000×g for 5 min at RT to allow phase separation. The supernatant was recovered, incubated with an equal volume of absolute isopropanol and then centrifuged. Pellet was re‐dissolved in an appropriate volume of nuclease‐free water.

### 2.4 Gene expression analysis

Treated seedlings or 4‐week‐old leaves were frozen in liquid nitrogen, homogenized with a MM301 Ball Mill (Retsch, Basel, Switzerland) for about 1 min at 24 Hz, and total RNA was extracted using the universal reagent for RNA isolation NucleoZol (Macherey‐Nagel) according to manufacturer's instructions. Total RNA (2 μg) was treated with RQ1 DNase (Promega), and cDNA was synthesized with the ImProm‐II™ Reverse Transcription System (Promega). qPCR was performed with a CFX96 Real‐Time PCR System (Bio‐Rad http://www.bio-rad.com). cDNA (25 ng of total RNA) was amplified in a 10 μL reaction mix containing 1X iTaq™ Universal SYBR® Green Supermix (Bio‐Rad) and 0.5 μM of each primer. Three technical replicates were analysed for each sample.

For each reaction, PCR efficiency (E) and Ct were calculated using the LinRegPCR software. Average expression level of each sample, relative to *UBQ5*, was determined using a modification of the Pfaffl method (Ruijter et al., 2013). Primer sequences used in this work are shown in Table S1.

Gene expression analysis was performed from at least three independent biological replicates, each composed by 20 seedlings or at least four adult leaves from different plants.

### 2.5 *Botrytis cinerea* growth and protection assay

Protection assays against *B. cinerea* were performed as previously described (Ferrari et al., 2007) with slight modifications. *Botrytis cinerea* (a kind gift of J. Plotnikova, Massachusetts General Hospital, Boston, MA) was grown for 10 to 15 d at 22°C under constant light on MEP media (malt‐agar 2% (w/v), peptone 1% (w/v) and micro‐agar 1.5% (w/v) until sporulation. Conidia were collected by flooding the plates with sterile water and filtered through Miracloth filter paper. Before plant inoculation, spores were suspended in 24 g L^−1^ potato dextrose broth (PDB) and incubated for 3 h at room temperature (RT) to allow uniform germination. Four‐week‐old Arabidopsis rosette were sprayed with water, OGs (200 μg ml^−1^) or flg22 (1 μM) 24 h before inoculation.

Inoculation was performed placing drops of spore suspension (5 *μ*L of 5 × 10^5^ conidiospores ml^−1^ per drop) on each side of the middle vein of leaves on plants. Lesion areas were determined by measuring necrotic tissues using ImageJ software 48 h after inoculation. The protection assays were repeated three times with consistent results.

### 2.6 Protein extraction and immunoblot analyses

*MAPKs phosphorylation*. Seedlings were frozen in liquid nitrogen, and crude extracts were prepared in phosphatase‐inhibiting buffer (50 mM Tris–HCl pH 7.5, 150 mM NaCl, 1 mM EDTA, 10 mM NaF, 1 mM Na_3_VO_4_, 1 mM Na_2_MoO_4_, 25 mM NaF, 10% (v/v) glycerol, 0.1% (v/v) Tween20, 1 mM dithiothreitol, 1 mM phenylmethylsulfonyl fluoride and 1X protease inhibitor cocktail P9599 (Sigma‐Aldrich). Equal amounts of proteins (40 μg) were resolved on 8% polyacrylamide gels and transferred onto nitrocellulose membranes using a Trans‐Blot Turbo apparatus (Bio‐Rad). Primary antibodies against phospho‐p44/42 MAP kinase (1:2000) and against MPK3 (1:2500) and MPK6 (1:10 000), in TBS‐T (Tris‐buffered saline, 0.1% Tween 20) containing 0.5% (w/v) BSA (bovine serum albumin), were used with a horseradish peroxidase–conjugated anti‐rabbit as secondary antibody (1:6000). Signal detection was performed using Clarity™ Western ECL substrate detection kit (Bio‐Rad) in the ChemiDoc MP imaging system (Bio‐Rad).

### 2.7 Detection of reactive oxygen species

Hydrogen peroxide produced by leaf discs was measured by a luminol‐based assay. Leaf discs (0.125 cm^2^) from 4‐week‐old plants were washed with sterile water every 30 min, for four times. One leaf disc per well was incubated overnight in sterile water in a 96‐well titre plate (Thermo Scientific NUNC). For elicitation with flg22, water was replaced with luminol solution (Sigma‐Aldrich; 30 μg ml^−1^) containing horseradish peroxidase (Sigma‐Aldrich; 20 μg ml^−1^) and flg22 (100 nM) or water as a control. For elicitation with OGs, discs were vacuum infiltrated with the OG solution (200 μg ml^−1^) or water as a control, for 2 min before addition of the luminol/peroxidase solution. ROS production was analysed for 40 min using a GloMax® Multi^+^ Detection System (Promega) and a signal integration time of 1 s. Luminescence was expressed in Relative Light Units (RLU). At least 12 leaf discs from four different plants were used for each biological replicate.

### 2.8 Determination of callose accumulation

Accumulation of callose was determined in 4‐week‐old rosette leaves 24 h after infiltration with OGs (50 μg ml^−1^) or flg22 (100 nM) and water as a control. After 24 h, 10 leaves from at least four independent plants for each treatment were cleared and dehydrated by boiling in 100% ethanol. Leaves were fixed in an acetic acid:ethanol (1:3) solution for 2 h, incubated for 15 min in 75% (v/v) ethanol, 15 min in 50% (v/v) ethanol and 15 min in 150 mM phosphate buffer (pH 8.0) and stained in 150 mM phosphate buffer (pH 8.0) containing 0.01% (w/v) aniline blue for 16 h at 4°C. After staining, leaves were mounted in 50% (v/v) glycerol and examined by UV epifluorescence microscopy (330–385 nm) (Nikon, Eclipse E200) using 10× magnification objective. The excitation was detected using a cooled charge‐coupled device CCD camera (DS‐Fi1C). Acquisition software is NIS‐Elements Advanced Research (Nikon). The number of callose deposits and the relative callose units were calculated using ImageJ.

### 2.9 FM4‐64 staining and brefeldin A treatment

Five‐day‐old proPCaP1:PCaP1‐GFP seedlings were treated with OGs (50 μg ml^−1^), flg22 (1 μM) or water for 30 min, then immediately stained with FM4‐64 (2 μM, Invitrogen, Molecular Probes) in 0.5X MS medium supplemented with 0.5% (w/v) sucrose for 5 min on ice and finally rinsed twice with cold medium. Imaging was performed within 10 min.

For brefeldin A (BFA) treatment, seedlings were first incubated for 60 min at RT in 1 ml of 0.5X MS liquid medium containing 50 μM BFA, then treated with OGs (50 μg ml^−1^), flg22 (1 μM) or water for 30 min and finally stained with FM4‐64 as described above.

### 2.10 Confocal laser‐scanning microscopy

An inverted spinning‐disk confocal microscope (CarvX, CrEST) was used for analyses. Imaging was performed using CFI Planfluo 40× (1.4 NA) oil immersion objective (NIKON) through a 70 μm pinhole disk set at 6000 rpm. GFP was excited using 473 nm laser light. Detection was performed using a cooled charge‐coupled device CCD camera (CoolSNAP HQ2, Photometrics) and omega band‐pass filters XF100‐2. The CCD camera, Z‐motor and confocal head were controlled by the Metamorph software (Molecular Devices).

Images of microdomain patterns in proPCaP1:PCaP1‐GFP seedlings were acquired using a ZEISS Spinning Disk Inverted microscope (Axio Observer with laser ablation unit) with 40× water immersion lens. The GFP was excited with a 488 nm argon laser and detected using a 525/50 bandwidth filter.

Endocytosis in the presence of FM4‐64 and BFA was imaged using a Zeiss LSM880 equipped with an Airyscan detector. PCaP1‐GFP and FM4‐64 imagings were performed using the 488 nm line of the argon laser (25 mW) and the 543 nm line of a He/Ne laser line (5 mW), respectively.

### 2.11 Image processing and data analysis

Microdomain quantification was performed on 5‐day‐old proPCaP1:PCaP1‐GFP seedlings by using ImageJ software, according to (Jarsch & Ott, 2015).

The amount of vesicles was expressed as the number of vesicles per image according to (Beck et al., 2012). Degree of colocalization was statistically analysed using Pearson's and Mander's coefficients by JACoP plugin embedded in the visualization and analysis software ImageJ version 1.45 s (Cordelières & Bolte, 2014; Schneider et al., 2012).

## 3 RESULTS

### 3.1 PCaP1 is required for OG‐induced priming

**FIGURE 1:**
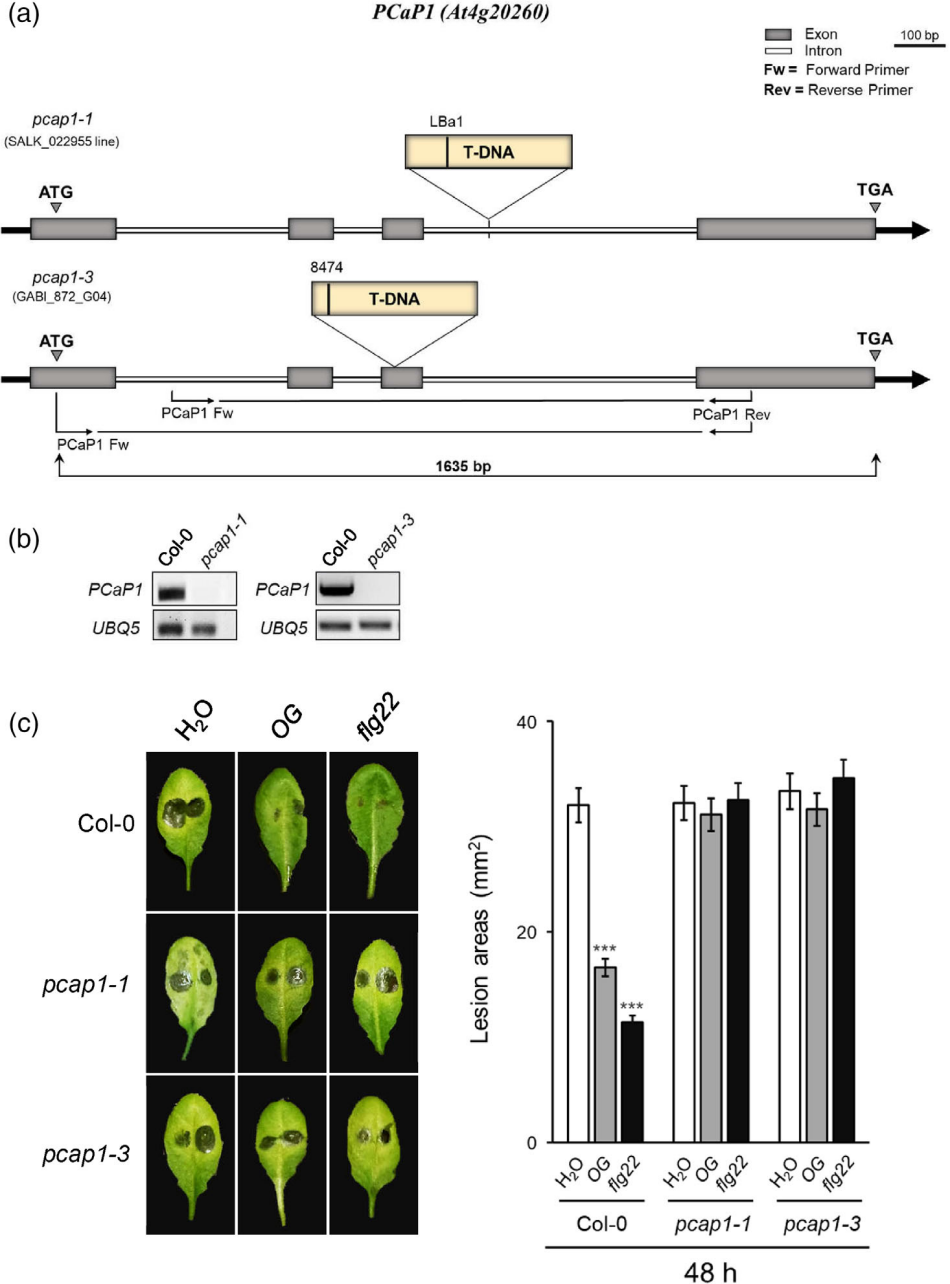
*pcap1* plants fail to display a reduction of lesion development after pre‐treatment with elicitors. (a) Position of the T‐DNA insertion in the allelic *pcap1‐*1 and *pcap1‐3* mutants. The coding exons and introns are represented in grey and in white, respectively. (b) Analysis of PCaP1 transcripts in seedlings of the wild type (Col‐0) and the two allelic *pcap1* mutants by RT‐PCR. UBQ5 was used as internal reference. (c) Induction of resistance to *B. cinerea* in wild‐type and *pcap1* mutants. Leaves were sprayed with OGs, flg22 or water 24 h before *B. cinerea* inoculation. Lesion areas were measured at 48 h after the inoculation. Values are means ± SE of at least 20 lesions. Asterisks indicate statistically significant differences between mutant and wild‐type lines according to Student's *t* test (*p < 0.05, **p < 0.01, ***p < 0.001) [Colour figure can be viewed at wileyonlinelibrary.com]

The identification of PCaP1 among the membrane proteins differentially phosphorylated within 10 min after treatment with OGs suggested its possible involvement in OG‐triggered immunity. In order to elucidate this, the effect of the loss of *PCaP1* in basal and induced resistance against the necrotrophic fungus *B. cinerea* was investigated in mutant lines defective for this gene. Two homozygous allelic T‐DNA insertion mutants (*pcap1‐1* and *pcap1‐3*) were identified (Figure 1a). Both mutants showed no expression of full‐length *PCaP1* transcripts (Figure 1b), indicating that they are null mutants, and no obvious morphological and developmental defects were observed (Figure S1).

Protection of Arabidopsis plants against *B. cinerea* is known to be induced by pre‐treatment with OGs or flg22 (Ferrari et al., 2007). This is a consequence of a ‘primed' state induced by the elicitors that make plants prone to respond more quickly and strongly to biotic and abiotic stresses (Conrath, 2011; Martinez‐Medina et al., 2016). To analyse the priming response in *pcap1* mutants, adult plants were sprayed with OGs, flg22 or water, and leaves were inoculated with *B. cinerea* spores 24 h after the treatment. While wild‐type plants pre‐treated with OGs or flg22 showed a reduced susceptibility compared to the water‐sprayed plants, elicitor pre‐treatment did not promote the same effect in both *pcap1* mutants, suggesting that PCaP1 is required for the elicitor‐induced priming (Figure 1c).

**FIGURE 2:**
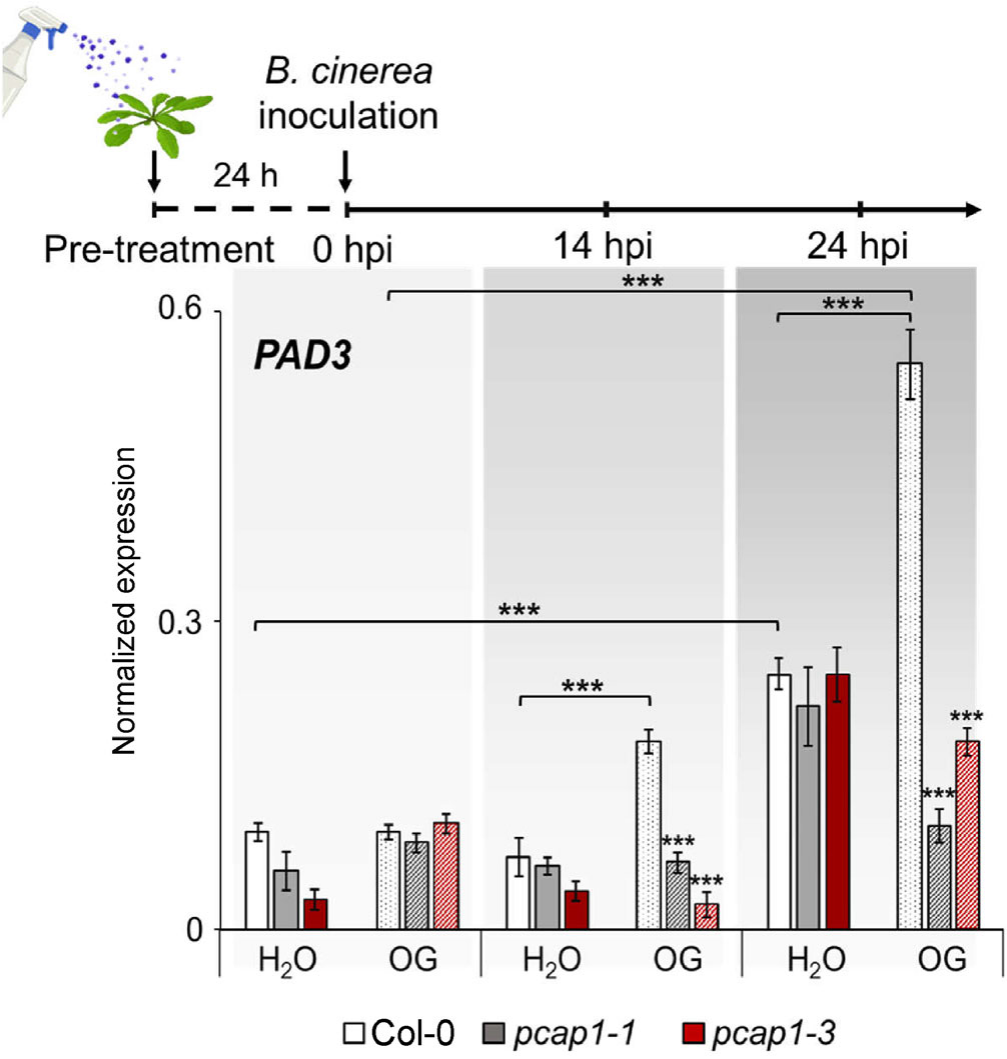
OG‐induced priming of the expression of the defense gene *PAD3* is abolished in *pcap1‐*mutant plants inoculated with *B. cinerea*. Upper panel, experimental design: leaves from mature plants were pre‐treated with water or OGs by spraying, and 24 h later were inoculated with *B.cinerea*. Leaves were collected at 0, 14 and 24 h post infection (hpi). Bottom panel, *PAD3* expression in the infected leaves. Wild type (WT; white bars), *pcap1‐1* (light‐grey bars) and *pcap1‐3* (dark‐red bars) rosette leaves were inoculated with *B. cinerea* after pre‐treatment with OGs, and total RNA was extracted at the indicated times. Expression of *PAD3* was analysed by qRT‐PCR using *UBQ5* as reference. Data are a mean ± SD of three independent experiments. Asterisks indicate statistically significant differences between mutant and wild‐type lines according to Student's *t* test (*p < 0.05, **p < 0.01, ***p < 0.001) [Colour figure can be viewed at wileyonlinelibrary.com]

To further investigate the basis of the lack of the elicitor‐induced protection in *pcap1*‐mutant plants upon infection with *B. cinerea*, we evaluated the expression of the defence‐related gene *PHYTOALEXIN DEFICIENT 3* (*PAD3, AT3G26830*), which encodes the enzyme involved in the last step of camalexin biosynthesis (Zhou et al., 1999). *PAD3* is required for OG‐induced protection against *B. cinerea* (Ferrari et al., 2007), and its expression during infection with *B. cinerea* is primed by pre‐treatment with OGs (Gravino et al., 2015). Levels of *PAD3* transcripts showed a moderate and similar increase at 24 h post infection (hpi) in both water‐sprayed (control) wild‐type and *pcap1*‐mutant plants (Figure 2). In wild‐type plants pre‐treated with OGs, *PAD3* transcripts significantly increased already at 14 hpi and, at 24 hpi, reached levels that were more than two‐fold higher compared to the water‐sprayed plants. In contrast, in *pcap1* mutants, pre‐treated with OGs, not only priming of *PAD3* expression did not occur but accumulation of *PAD3* transcripts was even reduced compared to the water‐pre‐treated plants (Figure 2). This result corroborates the conclusion that priming is impaired in *pcap1* mutants and further implies that the lack of protection against *B. cinerea* in the OG‐pre‐treated‐mutant plants could be at least in part ascribed to a defective priming of the expression of *PAD3*.

### 3.2 PCaP1 is required for OG‐induced late response and for restoration of the responsiveness to a second treatment with OGs

Next, we investigated the involvement of *PCaP1* in the response to elicitors. First, we analysed early responses such as OG‐induced phosphorylation of MPK3 and MPK6 and OG‐ and flg22‐induced accumulation of extracellular ROS in the *pcap1* mutants. Western blot analysis using an anti‐pTpY antibody that cross‐reacts with the double phosphorylated activation loop of MAPKs [TEY motif, (Ichimura et al., 2002)] revealed that levels of phosphorylated MPK3 and MPK6 were nearly comparable in the elicited mutants and wild‐type seedlings (Figure S2a). Also, elicitor‐induced apoplastic ROS production was not altered in the null‐mutant plants (Figure S2b).

**FIGURE 3:**
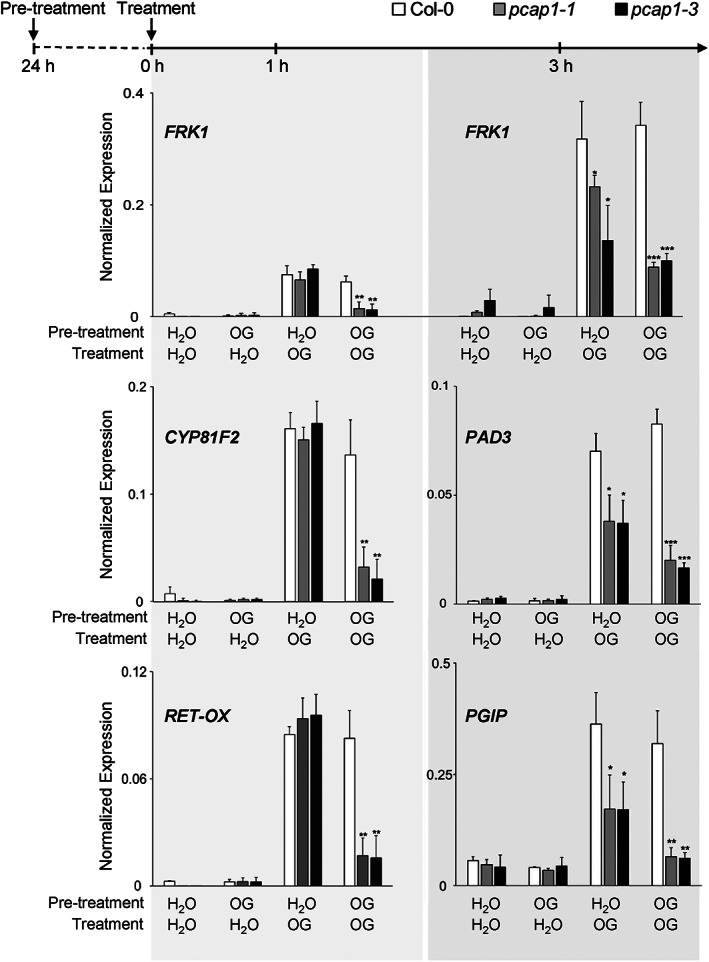
OG‐induced defense genes expression is decreased in *pcap1* mutants after pre‐treatment with OGs. Top, experimental design: seedlings were pre‐treated with either water or OGs on day 0, then after 24 h, again treated with water or OGs. Gene expression was measured 1 or 3 h after the second treatment. Bottom, *FRK1, CYP81F2, PAD3, RET‐OX* and *PGIP1* expression analysis in treated seedlings according to the experimental design is shown in the upper panel. Transcript levels are expressed as the gene/*UBQ5* ratio (normalized expression). Data are a mean ± SD of three independent experiments. Asterisks indicate statistically significant differences between mutant and wild‐type lines according to Student's *t* test (*p < 0.05, **p < 0.01, ***p < 0.001)

We further investigated whether *pcap1* mutants were affected in the response to OGs by analysing the induced expression of defense‐related genes *RET‐OX (At1g26380)*, *FRK1 (AT2G19190)* and *CYP81F2 (AT5G57220). RET‐OX*, renamed as *FOX1* (Boudsocq et al., 2010), encodes a berberine bridge enzyme–like protein involved in the conversion of indole‐cyanohydrin into the defense‐related metabolite indole‐3‐carbonyl nitrile (Rajniak et al., 2015). *FRK1* and *CYP81F2* encode an OG‐ and flg22‐induced receptor–like kinase and a cytochrome P450 monooxygenase involved in the biosynthesis of indole glucosinolate, respectively (Asai et al., 2002; Nafisi et al., 2007). These three genes are all considered as early‐MAMP‐ and DAMP‐induced genes (i.e., genes showing a maximal expression at 1 h after elicitation) (Denoux et al., 2008; Galletti et al., 2008; Gravino et al., 2017). In addition, we examined two late‐induced genes, typically showing maximal expression at 3 h or later after elicitation, that is, *PAD3* and *POLYGALACTURONASE‐INHIBITING PROTEIN 1* (*PGIP1, AT5G06860*), an OG‐ and *B. cinerea*–induced gene involved in basal resistance to this fungus (Ferrari et al., 2003). Transcript levels for these genes were examined upon an additional treatment with OGs for 1 h and 3 h performed 24 h after a pre‐treatment with either OGs (OG‐OG treatment; double treatment) or water (H_2_O‐OG treatment, single treatment). In seedlings subjected to H_2_O‐OG, no difference between wild‐type and mutant seedlings was observed at 1 h for *FRK1*, *CYP81F2* and *RETOX*; at 3 h, however, transcript levels for *FRK1, PAD3* and *PGIP1* were significantly reduced in both mutants (Figure 3). These results indicate that PCaP1 is required for full expression of the defense genes at a later stage but not at the early stage of the response to OGs. In seedlings subjected to the double OG treatment (OG‐OG), expression of all defense genes in the wild type at both 1 h and 3 h was not significantly different compared to the H_2_O‐OG treatment (Figure 3), suggesting that after 24 h, seedlings have totally recovered from the desensitization, known to occur upon two consecutive treatments with the same elicitor (refractory period <24 h) (Denoux et al., 2008). In the mutants, however, the expression of all analysed genes was reduced at both time points in the OG‐OG‐treated seedlings compared to H_2_O‐OG‐treated ones (Figure 3), a behaviour that may be interpreted as a slower recovery from desensitization in the mutants.

**FIGURE 4:**
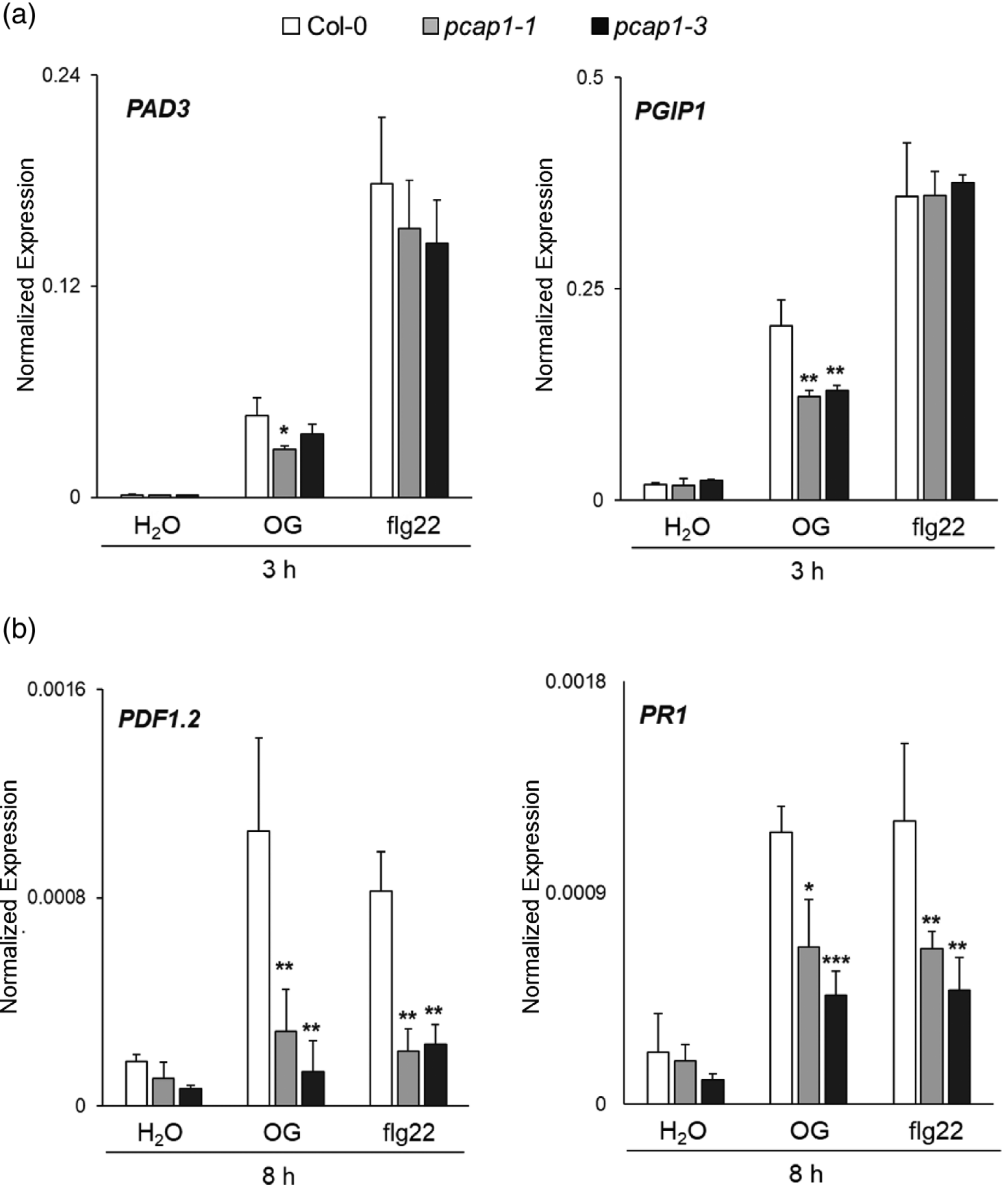
Expression of late defense‐response genes after elicitor treatment is reduced in *pcap1* mutants. Seedlings were treated with OGs or flg22 or water, as a control, and accumulation of *PAD3* and *PGIP1* transcripts was analysed after 3 h from the treatment (a), whereas *PDF1.2* and *PR1* transcripts after 8 h from the treatment (b). Transcript levels are expressed as the gene/*UBQ5* ratio (normalized expression). Data are a mean ± SD of three independent experiments. Asterisks indicate statistically significant differences between mutant and wild‐type lines according to Student's *t* test (*p < 0.05, **p < 0.01, ***p < 0.001)

**FIGURE 5:**
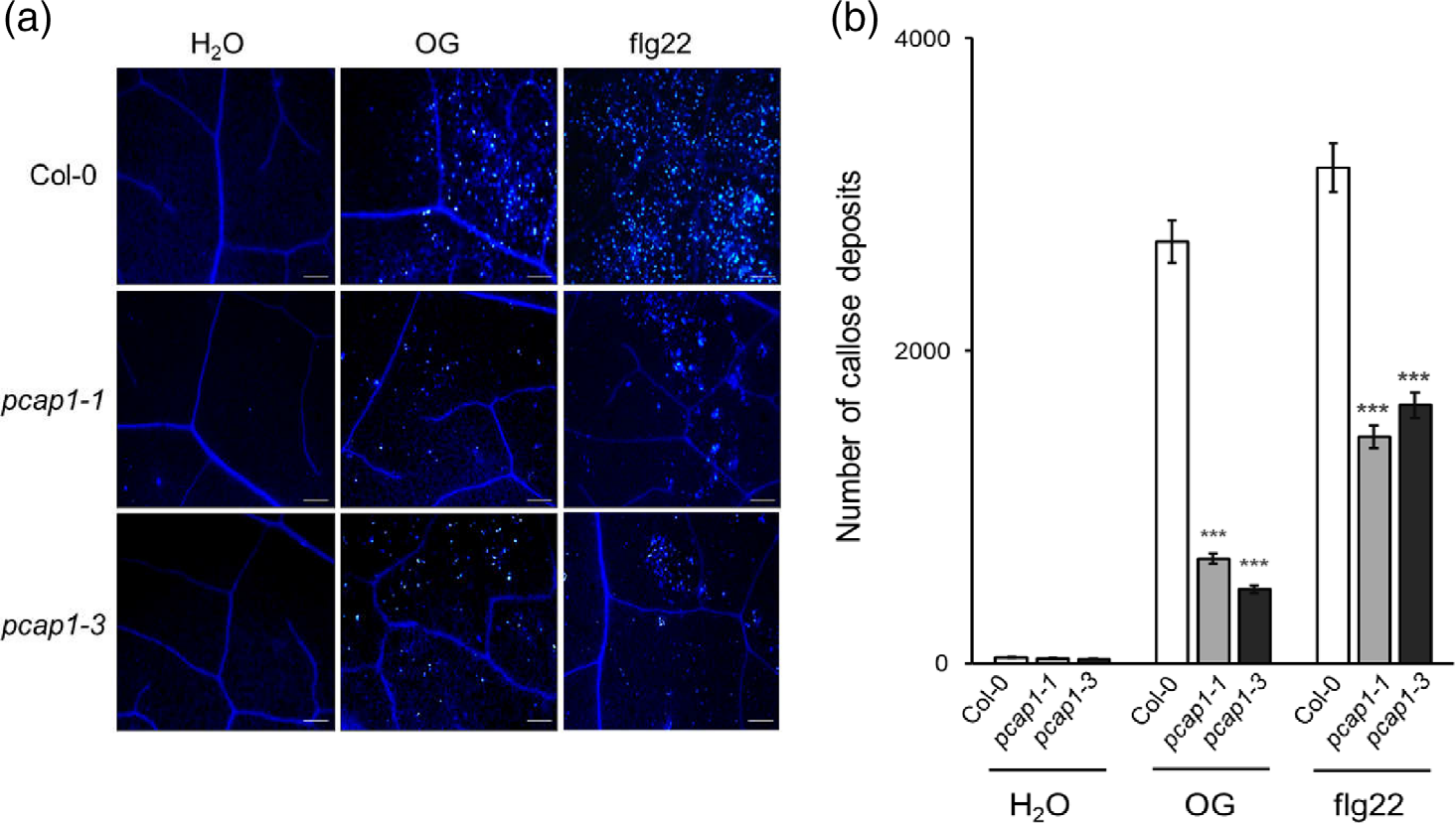
Callose deposition induced by elicitor infiltration is strongly reduced in *pcap1* mutants. (a) Leaves from Arabidopsis wild‐type and *pcap1* plants were infiltrated with water, OGs or flg22, and the excised leaves were stained 24 h later with aniline blue for callose visualization. Images show representative leaves for each treatment. Scale bar = 0.1 mm (10× magnification). (b) Number of callose depositions quantified as the number of individual depositions per unit of leaf surface infiltrated with elicitors or water. Data are means ± SE (n = 6) of four microscopic fields (0.1 mm^2^ for each). Asterisks indicate statistically significant differences between mutant and wild‐type lines according to Student's *t* test (*p < 0.05, **p < 0.01, ***p < 0.001) [Colour figure can be viewed at wileyonlinelibrary.com]

Given the observed patterns, we decided to additionally study late responses in the mutants. The expression of *PDF1.2* (*plant defensin 1.2, AT5G44420*) and *PR1* (*pathogenesis‐related 1 protein, AT2G14610*), two marker genes that are induced 8 h after elicitation (Gravino et al., 2017), was analysed in seedlings upon treatment with OGs and flg22, showing a significant reduction in the mutants (Figure 4b). To test whether these different gene expression patterns correlate to later defense responses, we investigated whether the deposition of callose, a well‐known late response to MAMPs and DAMPs and a cellular marker for immunity (Denoux et al., 2008; Gomez‐Gomez et al., 1999) is altered in the mutants. For this, callose accumulation upon infiltration with OGs or flg22 was analysed by aniline blue staining (Figure 5a). Indeed, leaves of both *pcap1* mutants displayed strongly reduced callose accumulations compared to wild‐type leaves in response to both elicitors, indicating that *PCaP1* is required for this response (Figure 5b). Since *GSL5/PMR4 (AT4G03550)* encodes the callose synthase (CalS) responsible for pathogen‐induced callose deposition, we analysed the expression of this gene in the leaves of both wild‐type and *pcap1*‐mutant plants 24 h after OG infiltration. In line with the findings above, a significant reduction of *PMR4* gene expression was observed in *pcap1* plants compared to the wild type (Figure S3 **)**.

Taken together, our observations indicate that PCaP1 is required for late defense responses to elicitors and for the recovery of full responsiveness to OGs after an elicitor treatment, therefore showing a role of the protein in PTI.

### 3.3 PCaP1 localizes into PM microdomains and is rapidly internalized in response to OGs

**FIGURE 6:**
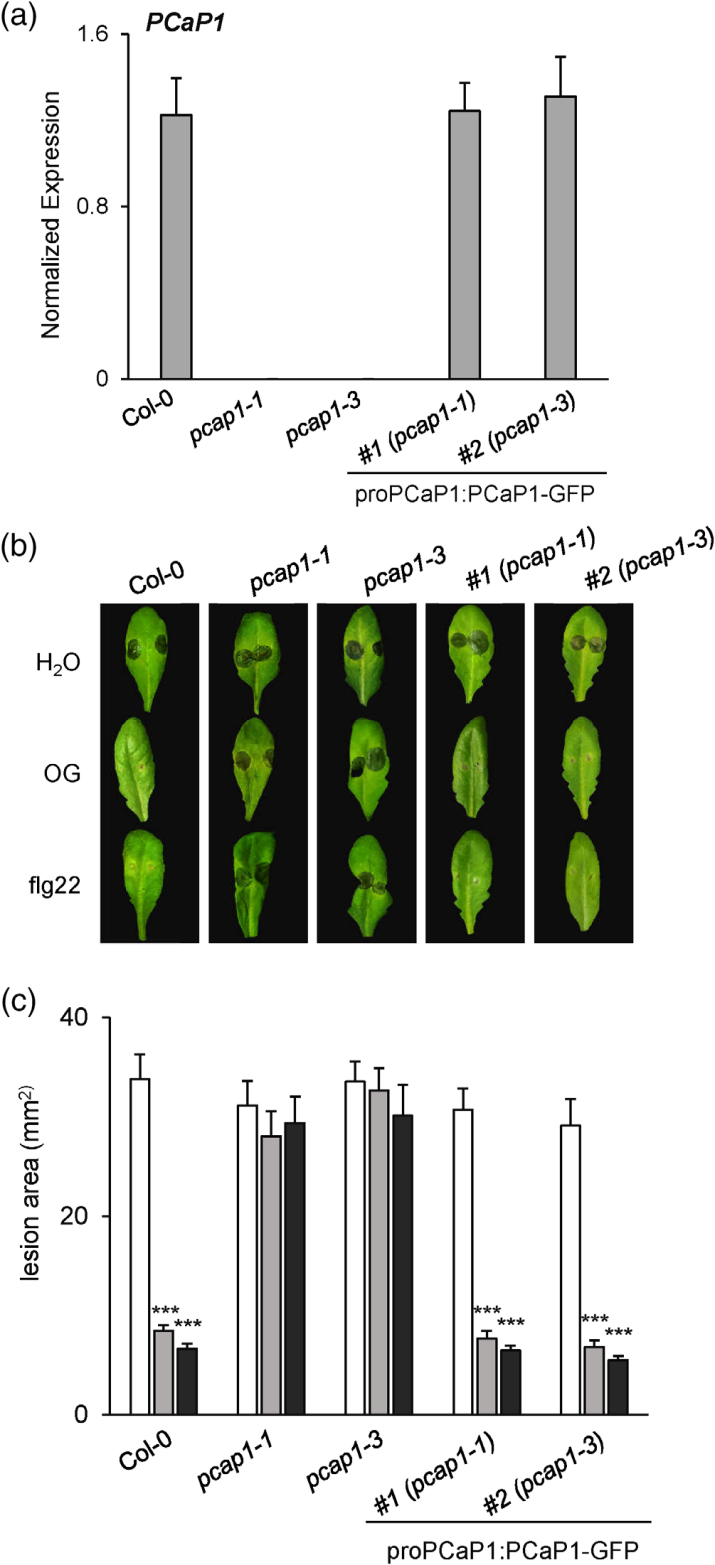
Complementation of *pcap1* mutants in Arabidopsis. (a) Transcript levels of PCaP1 in Wt, *pcap1‐1* and *pcap1‐3* null mutants and two independent complemented lines (proPCaP1:PCaP1‐GFP/*pcap1‐1* and proPCaP1:PCaP1‐GFP/*pcap1‐3*) expressed as the gene/*UBQ5* ratio (normalized expression). (b) Induction of resistance to *B. cinerea* infection in Arabidopsis wild type, null mutants and complemented lines subjected to spray pre‐treatment with water, OGs or flg22 24 h before inoculation; pictures were taken at 48 hpi. (c) Lesion area analysis (mm^2^) of the same plants shown in (b). Results are average ± SE (n = 20 lesions). Asterisks indicate statistically significant differences between lines according to Student's t test (*p < 0.05, **p < 0.01, ***p < 0.001) [Colour figure can be viewed at wileyonlinelibrary.com]

PCaP1 has been identified, after subcellular fractionation, as a protein stably associated with the PM (Ide et al., 2007; Mattei et al., 2016). Thus, we decided to further investigate the distribution of PCaP1 on the PM by using transgenic plants expressing a GFP‐tagged PCaP1 under control of its native promoter (proPCaP1:PCaP1–GFP) (Nagata et al., 2016). The PCaP1‐GFP fusion protein localized at the PM (Figure S4), in accordance with previous reports (Nagata et al., 2016). We assessed the capability of the PCaP1‐GFP fusion protein of restoring a normal elicitor‐induced protection against *B. cinerea* in the two allelic *pcap1* mutants. Both *pcap1* mutants homozygous for the proPCaP1:PCaP1–GFP transgene showed levels of *PCaP1* transcripts comparable to wild‐type plants **(**Figure 6a **)** and restored wild‐type elicitor‐induced protection against *B. cinerea* (Figure 6b,c), demonstrating that the PCaP1‐GFP fusion is functional.

**FIGURE 7:**
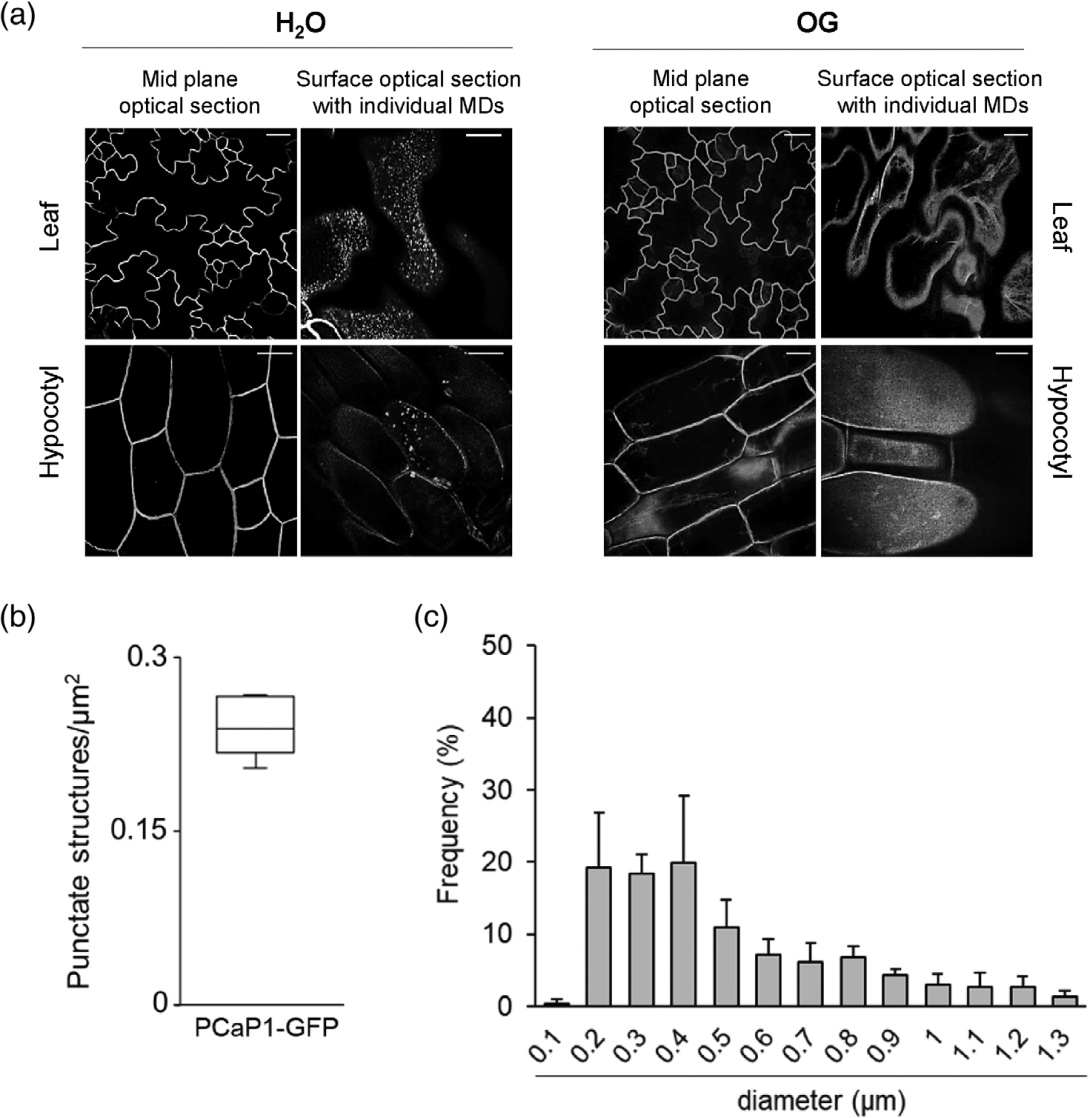
PCaP1 localizes in plasma membrane microdomains. Confocal laser scanning microscopy at the cell surface of cotyledons and hypocotyls in water‐treated and OG‐treated proPCaP1:PCaP1‐GFP seedlings (a); scale bar = 20 μm. (b) Average density of the PCaP1 punctate structures, also referred to as microdomains, observed in (a) and expressed as the number of punctate structures per μm^2^ cell surface. No microdomains were observed upon OG treatment. (c) Frequency of microdomain diameter size (μm) measured in seven independent cells. Data are mean ± SD

The precise distribution of PCaP1 in the PM was examined in vivo in young proPCaP1:PCaP1‐GFP seedlings by confocal laser scanning microscopy. PCaP1 fluorescence was found to be heterogeneously distributed on the PM in both cotyledons and hypocotyls (Figure 7a) and organized in densely packed and punctate structures (0.26 ± 0.016 punctate structures per μm^2^ of cell surface) (Figure 7b), with a diameter ranging from 0.2 to 1.3 μm (Figure 7c). The distribution pattern and the size of these structures were similar to other microdomain‐localized proteins as described in *Arabidopsis* and *Nicotiana benthamiana* (Jarsch et al., 2014), suggesting that PCaP1 is organized in membrane microdomains. It should be noted that this accumulation dynamically changed as PCaP1‐positive putative microdomains nearly disappeared after a 30 min treatment with OGs in both organs, and fluorescence appeared to be spread uniformly over the cell surface (Figure 7a).

**FIGURE 8:**
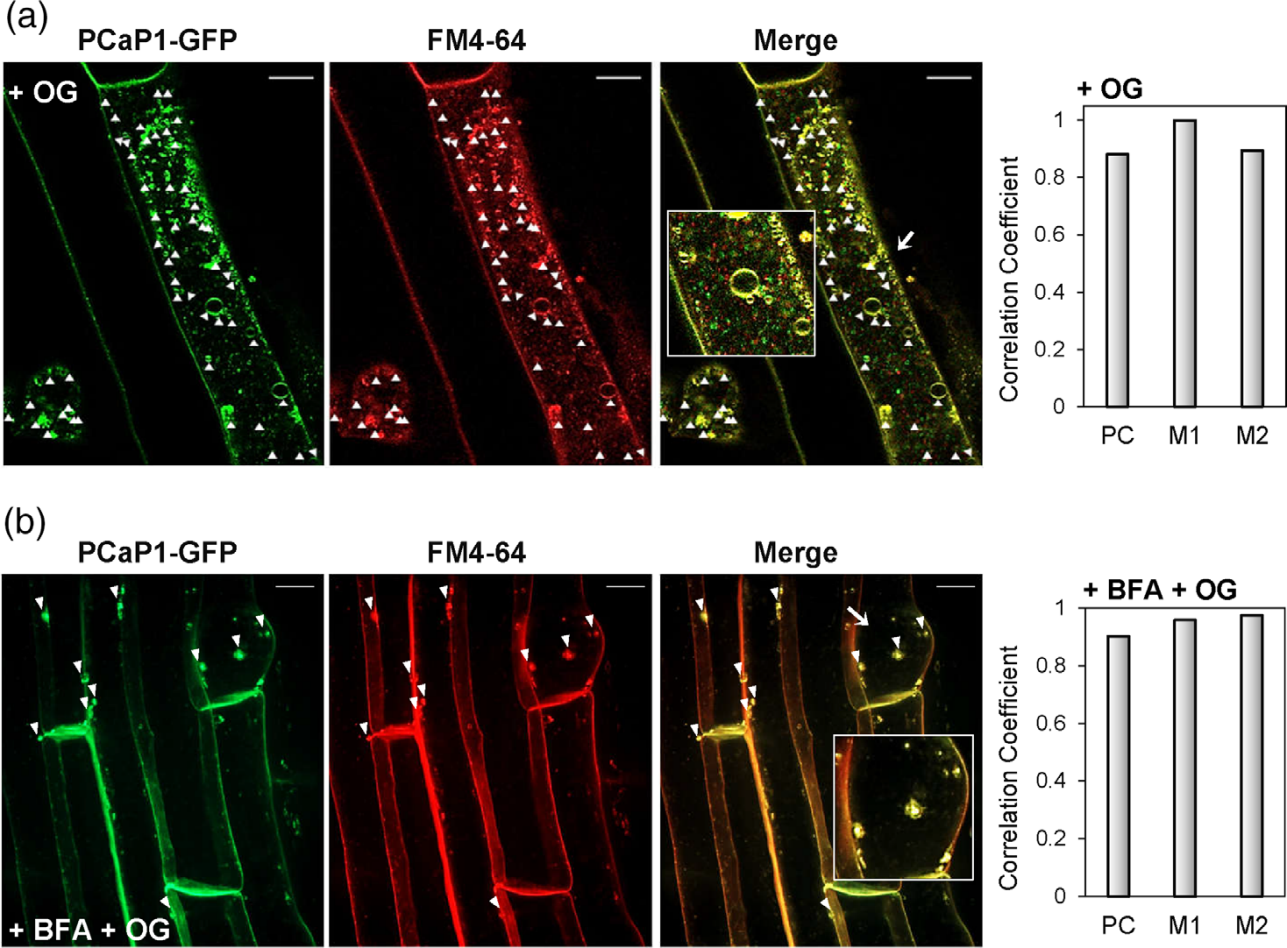
PCaP1‐GFP is rapidly internalized in endocytic vesicles in response to OGs. Confocal laser scanning microscopy on roots of proPCaP1:PCaP1‐GFP seedlings, stained with the endocytic tracer FM4‐64 upon treatment with (a) OGs and (b) BFA + OGs. In (a), colocalization of PCaP1‐GFP and FM4‐64 was observed at the vesicle membranes, whereas in (b), colocalization of PCaP1‐GFP and FM4‐64 was observed in the BFA compartments. [FM4‐64 labelling: red, PCaP1‐GFP: green, merged channels: orange]. Insets: magnification of areas of interest. Scale bar = 10 μm. In both (a) and (b), the degree of colocalization between the green and red signals was statistically analysed and expressed with Pearson's correlation coefficient (PC) and the Mander's colocalization coefficients M1 and M2 (in each panel, graph on the right). M1 represents the fraction of PCaP1‐GFP (green signal) overlapping with FM4‐64 (red signal). M2 represents the fraction of FM4‐64 (red signal) overlapping with the PCaP1‐GFP (green signal) [PC = ‐1, complete anti‐colocalization; PC = 0, non‐colocalization; PC = 1, complete colocalization] [Colour figure can be viewed at wileyonlinelibrary.com]

Moreover, cell cortex optical sections of epidermal root cells revealed a fluorescence associated with numerous vesicles (Figure S5a,b), the abundance of which significantly increased after a 30 min treatment with OGs or flg22 (Figure S5c,d) and returned to basal levels already after 1 h. The nature of PCaP1‐containing vesicles was investigated by co‐localization analyses using the tracer FM4‐64, commonly used to study the dynamic process of endocytosis (Rigal et al., 2015). In proPCaP1:PCaP1‐GFP seedlings treated with OGs, a high degree of co‐localization between the FM4‐64 signal and the PCaP1‐GFP fluorescence‐positive vesicles was detected **(**Figure 8a **),** indicating that PCaP1‐GFP is rapidly internalized in endocytic vesicles in response to OGs. Endocytosis induced by OGs, however, is not altered in *pcap1*‐mutant seedlings, since the number of FM4‐64 positive vesicles was comparable between the wild type and the mutants (Figure S6).

To further analyse the observed patterns, PCaP1‐GFP/FM4‐64 colocalization analysis was additionally performed after treatment with OGs in the presence of brefeldin A (BFA). This fungal inhibitor causes aggregation of trans‐Golgi network (TGN), endosomal and Golgi material in large intracellular bodies, named BFA compartments (Langhans et al., 2011). These compartments are known to accumulate material of endocytic origin (Rosquete et al., 2018; Viotti et al., 2010). OG treatment in the presence of BFA resulted in PCaP1‐GFP accumulation in BFA compartments, where it co‐localized with the membrane marker dye FM4‐64 (Figure 8b). Taken together, these results indicate that PCaP1‐GFP re‐localizes to the TGN by endocytosis upon OG treatment.

## 4 DISCUSSION

In this work, we investigated the importance of PCaP1 during OG‐triggered immune responses by analysing two Arabidopsis homozygous loss‐of‐function *pcap1* allelic mutants (Figure 1 a,b). We uncovered that PCaP1, which is phosphorylated 10 min after treatment with OGs (Mattei et al., 2016), indeed plays a role in *A. thaliana* immunity and in the response to a DAMP (OGs) and an MAMP (flg22). Protection against *B. cinerea* induced by both elicitors is defective in both allelic mutants, although basal resistance against this fungus is not affected (Figure 1c). Consistent with this result, the expression of *PAD3* during infection with *B. cinerea* is not primed in the mutants upon pre‐treatment with elicitors; in fact, it was even lower than in control water‐pre‐treated plants (Figure 2). Moreover, a more rapid decrease of the induced expression of the early marker gene *FRK1* occurs in mutant seedlings treated with OGs or flg22 compared to wild‐type (Figure 3). A similar reduction was also observed in the expression of late genes, such as *PAD3, PGIP1*, *PDF1.2* and *PR1* (Figure 4) Intriguingly, only the OG‐induced but not the flg22‐induced expression of *PGIP1* was affected in the *pcap1* mutants, suggesting that distinct pathways lead to *PGIP1* upregulation in response to OGs or flg22 (Figure 4). The lack of PCaP1 also leads to a defective recovery of full responsiveness to a second treatment with OGs in the mutants, pointing towards an involvement of the protein in the restoration of responsiveness to OGs upon consecutive treatments (Figure 3). In adult plants, callose deposition, another late elicitor‐induced response, is also impaired in *pcap1* mutants (Figure 5), due to a reduction of the induced expression of the callose synthase–encoding gene *PMR4* (Figure S3). Early responses, such as activation of MPKs, expression at 1 h of *RETOX* and accumulation of apoplastic H_2_O_2_, known to be mediated by the NAPDH oxidase RBOHD (Galletti et al., 2008), instead were not affected (Figure S2). The observation of a normal response of the MAPK cascade in spite of an altered expression of defense genes is not unprecedented (Gravino et al., 2015; Savatin et al., 2014) and may indicate an action of PCaP1 either downstream of MAPK cascade or in an independent parallel pathway; however, taken together, our data point to a role of PCaP1 in the late but not in the early responses to OGs and flg22.

The increase of the intracellular Ca^2+^ levels is a primary event in immunity induced by OGs and MAMPs (Chandra & Low, 1997; Messiaen & Van Cutsem, 1994; Navazio et al., 2002; Ranf et al., 2012; Tena et al., 2011) and likely modulates the function of PCaP1, through mechanisms that need to be elucidated. Intracellular Ca^2+^ levels affect the response to OGs and flg22 also through the action of CDPKs, since the simultaneous loss of *CPK5*, *CPK6* and *CPK11* affects both basal and elicitor‐induced resistance to *B. cinerea* (Gravino et al., 2015). Their loss does not affect the induced ROS production mediated by RBOHD, although the complex regulation of the enzyme has been shown to involve also CDPKs for activation (Kadota et al., 2015; Wang et al., 2018). Instead it does affect the duration, but not the onset, of the OG‐ and flg22‐induced expression of the early‐induced genes *CYP81F2*, *FRK1* and *PHI‐1* (Gravino et al., 2015). Unlike the *pcap1* mutants, however, callose deposition is not affected by the loss of *CPK5*/*CPK6*/*CPK11* (Gravino et al., 2015). These results show that these CDPKs, like PCaP1, play a role in a secondary phase of the response to elicitors but, at least in part, in independent pathways.

In this work, we not only confirmed the localization of PCaP1 at the PM but demonstrate that the protein is organized in punctate structures on the cell surface under basal conditions, while this pattern disappears upon OG treatment (Figure 7a). Interestingly, nanodomain association has also been demonstrated for a symbiosis‐related DREPP protein in *Medicago truncatula*, although nanodomain association of DREPP was induced in the presence of a rhizobial signal (Su et al., 2020). These data are in accordance with the observation that PCaP1 is recovered from sterol‐rich membrane fractions, the so‐called ‘detergent‐resistant membranes' (DRMs) (Kierszniowska et al., 2009). Indeed, membranes are dynamically organized as a heterogeneous mosaic of small regions, termed nano‐ and microdomains (Ott, 2017), with varying lipid composition and properties and a defined protein content (Gronnier et al., 2018; Hemsley, 2015; Konrad & Ott, 2015). The association of PCaP1 to the PM and its specific localization have been shown to depend on the N‐terminal myristoylation site (Maurer‐Stroh et al., 2002; Nagasaki et al., 2008; Su et al., 2020) which may confer the capability of clustering in PM microdomains. N‐myristoylation can increase the membrane affinity of polypeptides and assist their targeting to membrane domains, as in the case of animal flotillins (Neumann‐Giesen et al., 2004). The functional significance of PCaP1 localization in microdomains, however, remains still unknown: it may reflect the sites of cytoskeleton‐PM interaction as suggested for the *Medicago* DREPP protein, that is recruited into functional membrane nanodomains and triggers microtubule fragmentation during symbiotic infection (Su et al., 2020). In Arabidopsis PCaP1, the same region of the protein is responsible for both membrane and microtubule interaction (Li et al., 2011), suggesting that the protein could not bind to the plasma membrane and microtubule simultaneously.

Notably, the localization of PCaP1 in membrane microdomains nearly disappears 30 minutes after OG treatment (Figure 7a), while it appears in endocytic vesicles of heterogeneous size, some of which may represent endosomes (Figure 8a). Endocytosis is known as a mechanism for cellular desensitization by removing ligand‐bound receptors from the PM; it remains to be tested whether the vesicles observed in our analyses also harbour components of the elicitor perception machinery and whether recruitment of PCaP1 in endosomes plays a role in turning off OG signalling. Since the loss of *PCaP1* does not affect endocytosis per se, our results, together with the reduced response of *pcap1* mutants to a second OG treatment, point to a more specific role of PCaP1 and likely its turn‐over in the sensing/transduction response to elicitors.

PCaP1 is rapidly phosphorylated upon treatment with both OGs and flg22 (Mattei et al., 2016; Rayapuram et al., 2014); however, the features and role of the phosphorylation state of PCaP1 on the induction of defence responses and on its localization are not yet known. The impact of phosphorylation/dephosphorylation on the affinity of proteins to membrane environments has been shown for microdomain markers belonging to the REMORIN (REM) protein family. Remorins harbour the majority of phosphorylation sites in the N‐terminal intrinsically disordered region (Marin & Ott, 2012). The phosphorylation of Remorin 1.3 (REM1.3) at its N‐terminal domain, mediated by CPK3 in a calcium‐dependent manner, modulates its dynamic localization in PM nanodomains and plasmodesmata (PD) and is crucial for the regulation of callose deposition at PD pit fields and the restriction of viral cell‐to‐cell movement (Perraki et al., 2018). Of note, also REM1.3 has been found among the early phosphorylated proteins upon treatment with OGs (Farmer et al., 1990; Mattei et al., 2016); however, whether phosphorylation of PCaP1 involves CDPKs is not yet known. Moreover, it has to be elucidated whether elicitor‐induced phosphorylation of PCaP1 changes its affinity to PI(3,5)P_2_ and PI(4,5)P_2_, which, although representing the smallest fraction of total PI pool of the PM, are known to recruit membrane proteins involved in several essential cellular processes, including the regulation of cellular trafficking and actin polymerization (Boss & Im, 2012; Noack & Jaillais, 2017; Shisheva, 2008; Tan et al., 2015). PI(3,5)P_2_ and PI(4,5)P_2_ are also dynamically up‐regulated during plant infection and in response to stress, respectively (Dove et al., 1997; Meijer et al., 1999; Qin et al., 2020), indicating a role in cellular homeostasis and in adaptation. It is therefore plausible that PI(4,5)P_2_ and PI(3,5)P_2_ might orchestrate the endocytotic turnover to modulate and maintain the presence of proteins on the PM during the immune response, also through the interaction with membrane proteins, including PCaP1.

**FIGURE 9:**
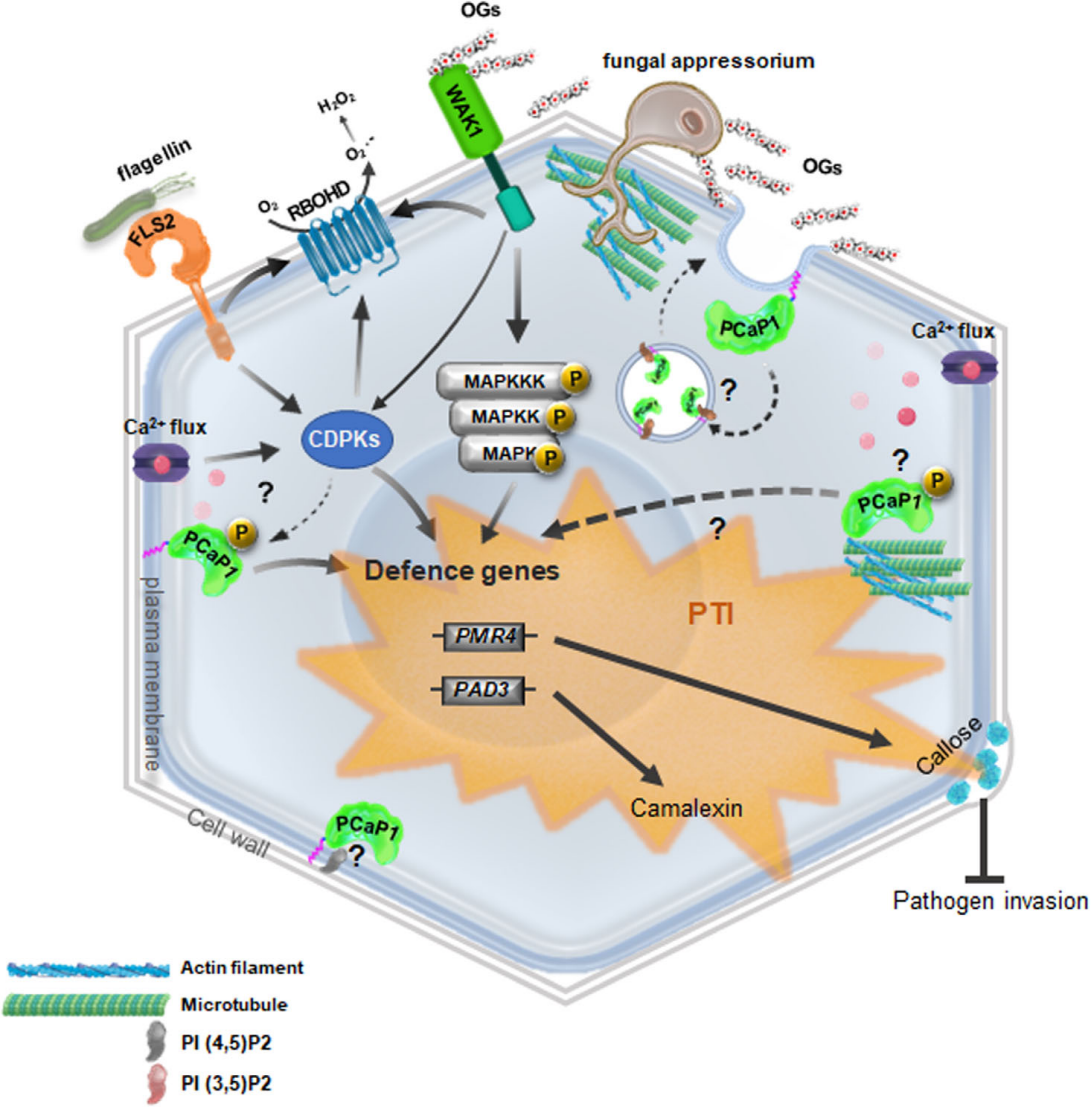
Model of the role of PCaP1 in *Arabidopsis thaliana* immunity and response to elicitors. PAMPs (e.g., flg22) and DAMPs (e.g., OGs) induce a ‘primed' state in the plant, and PCaP1 is required for this response, in particular for the expression of the late defence‐related genes, elicitor‐induced protection against *B. cinerea* and callose accumulation. PCaP1 is unlikely to be directly involved in the transduction of the Ca^2+^ signal that leads to early responses but may participate in the dynamic rearrangement and disruption of the cytoskeleton during pathogen attack. PI(3,5)P_2_ and PI(4,5)P_2_ may modulate PCaP1 endocytotic turnover for maintaining it on the PM during the immune response. FLS2: FLAGELLIN‐SENSITIVE 2 protein specific receptor of flagellin (flg22) [WAK1: CELL WALL‐ASSOCIATED KINASE 1 protein (*mediates the perception of OGs*), RBOHD: NADPH/respiratory burst oxidase protein D, PAD3: PHYTOALEXIN DEFICIENT 3 (*required for camalexin production*), PMR4: POWDERY MILDEW RESISTANT 4 (*required for wound and papillary callose formation*)] [Colour figure can be viewed at wileyonlinelibrary.com]

We propose a model for the action of PCaP1 **(**Figure 9 **),** depicting the possible links between PCaP1 phosphorylation, calcium signalling, the regulation of cytoskeleton organization and defense priming. Although it is not known yet if PCaP1 is phosphorylated by CDPKs in a calcium‐dependent manner, the protein may link calcium signalling to the dynamic rearrangement and disruption of the cytoskeleton during pathogen attack. Plant cells often respond to diverse microbes and elicitors by increasing abundance or bundling of actin filaments (Henty‐Ridilla et al., 2013; Takemoto & Hardham, 2004; Thomas, 2012). The involvement of the actin–myosin system in the internalization and trafficking of some PM receptors, for example, FLS2, has been demonstrated (Beck et al., 2012), and treatment with actin‐depolymerizing drugs triggered resistance to pathogens in Arabidopsis plants by inducing an increase in salicylic acid levels (Leontovyčová et al., 2019); however, the molecular machinery that senses and transduces immune signalling to actin cytoskeleton remodelling and vesicle dynamics is not fully known. Actin rearrangements conferred by actin depolymerizing factors/cofilins (AC) regulated through cycles of phosphorylation and dephosphorylation, also facilitate effector‐mediated internalization of bacterial pathogens into host mammalian cells (Dai et al., 2004). PCaP1 dynamics might be also regulated by the presence of PI(3,5)P_2_ and PI(4,5)P_2_, known to interact with membrane proteins in response to stress during plant infection, suggesting a modulation of PCaP1 endocytotic turnover for maintaining it on the PM during the immune response.

Further studies are still required to elucidate how phosphorylation induced by OGs or flg22 may redirect PCaP1 from membranes to cytosol or other compartments to regulate actin filament turnover and trafficking, and more downstream, gene expression and priming independently from MAPK activation and ROS production.

## AUTHOR CONTRIBUTION

MG, GDL and BM designed the experiments and analysed data. MG performed the experiments and wrote the manuscript draft. SF, LM and TO contributed to design and to perform the experiments. NT and MM provided PCaP1‐GFP plants. BM and GDL supervised the research. BM and GDL wrote the manuscript in its final version. All authors have approved the final manuscript.

## Supporting information

**Figure S1** Phenotype of wild‐type and *pcap1* null‐mutant plants.

**Figure S2***PCaP1* is not required for elicitor‐induced early defence responses.

**Figure S3** The induction of *PMR4* in response to OGs is reduced in *pcap1‐*mutant plants.

**Figure S4** Plasma membrane localization of PCaP1–GFP.

**Figure S5** Quantitative imaging analysis of OG‐ and flg22‐induced endocytosis in proPCaP1:PCaP1‐GFP seedlings.

**Figure S6** Confocal microscopy micrographs of root epidermal cells in response to OGs in null mutants.

**Table S1**. Primers used in this work.

## Data Availability

All data generated or analysed during this study are included in this published article [and its supplementary information files].
