# Supplementary figures and images for: The plasma membrane–associated Ca2+ ‐binding protein, PCaP1, is required for oligogalacturonide and flagellin‐induced priming and immunity
Source: Plant Cell Environ. 2021 Jun 30;44(9):3078–93. doi: 10.1111/pce.14118 (PMC8457133; doi:10.1111/pce.14118)

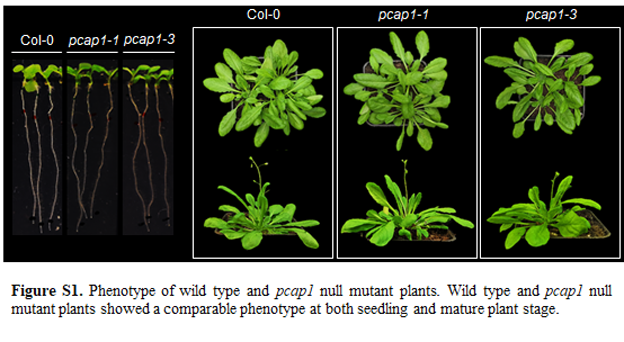

Supplement: Supplementary file 1 — Figure S1 Phenotype of wild‐type and pcap1 null‐mutant plants. [file PCE-44-3078-s007.tif]

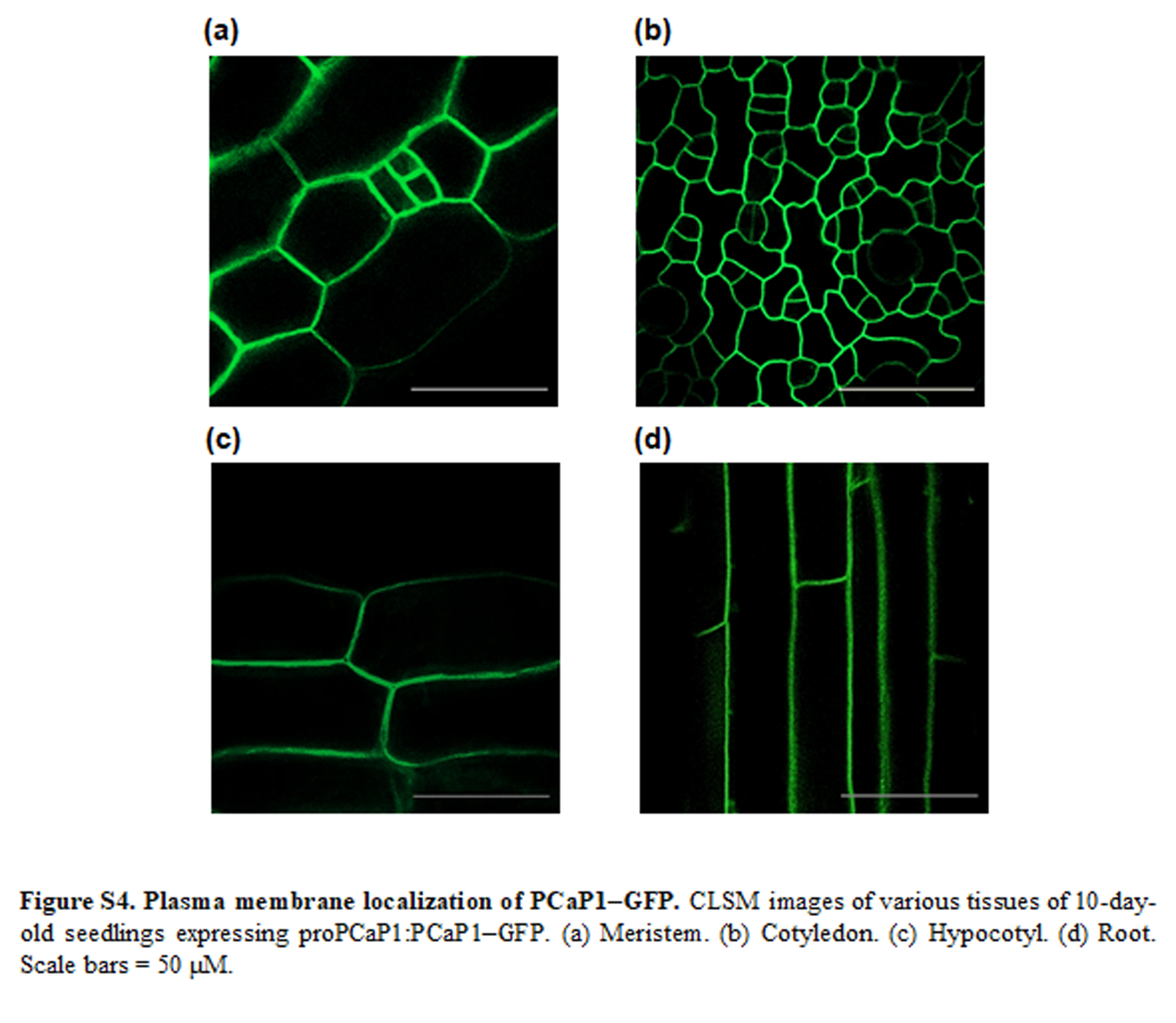

Supplement: Supplementary file 4 — Figure S4 Plasma membrane localization of PCaP1–GFP. [file PCE-44-3078-s003.tif]
