# Supplementary material for: The plasma membrane–associated Ca2+ ‐binding protein, PCaP1, is required for oligogalacturonide and flagellin‐induced priming and immunity
Source: Plant Cell Environ. 2021 Jun 30;44(9):3078–93. doi: 10.1111/pce.14118 (PMC8457133; doi:10.1111/pce.14118)
Supplement: Supplementary file 2 — Figure S2PCaP1 is not required for elicitor‐induced early defence responses. [file PCE-44-3078-s002.pdf]

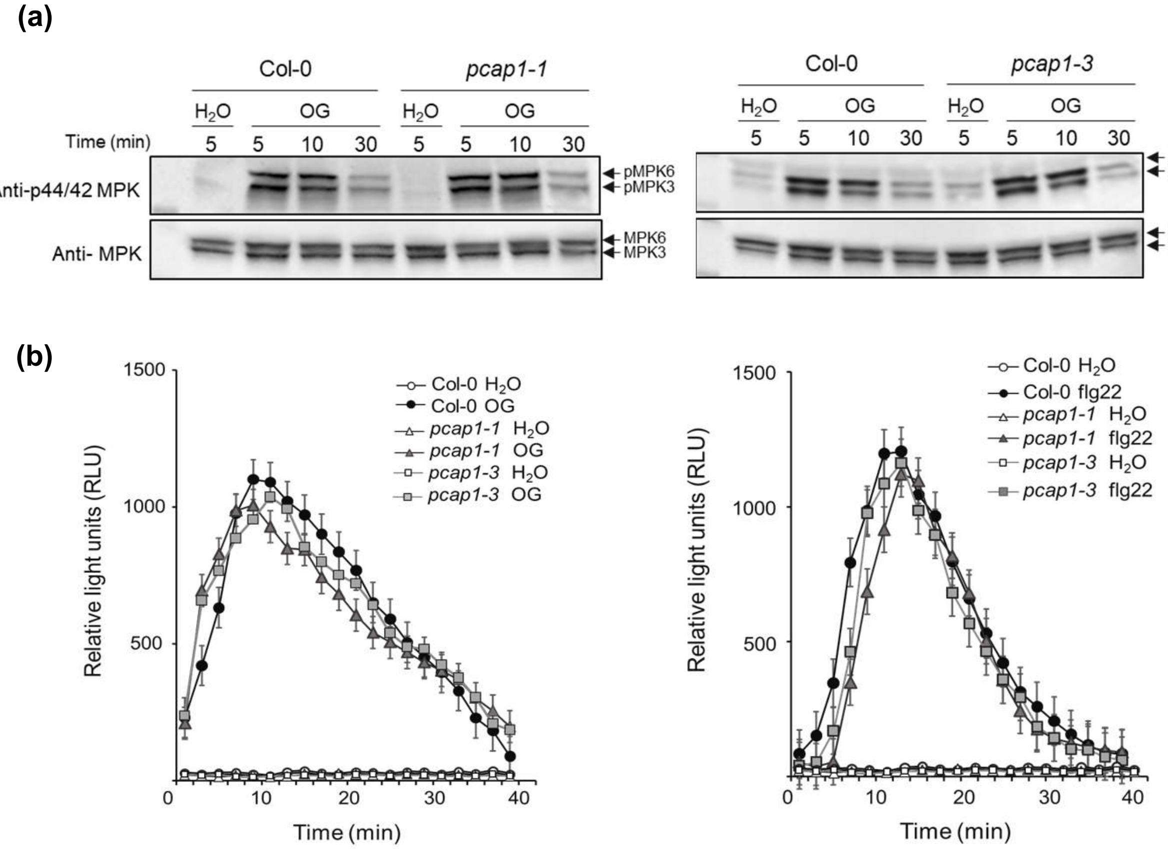

Figure S2. *PCaP1* is not required for elicitor-induced early defence responses. (a) Phosphorylated MPKs were detected by immunoblot using an α-p44/p42 antibody. Native MPKs were detected by immunoblot using an α-MPK3/6 antibody. (b) ROS production was measured in wild-type and *pcap1* null mutant leaf discs after elicitation with water, OGs or flg22. Data are expressed as mean ± SD (n = 12).
