# Supplementary material for: The plasma membrane–associated Ca2+ ‐binding protein, PCaP1, is required for oligogalacturonide and flagellin‐induced priming and immunity
Source: Plant Cell Environ. 2021 Jun 30;44(9):3078–93. doi: 10.1111/pce.14118 (PMC8457133; doi:10.1111/pce.14118)
Supplement: Supplementary file 3 — Figure S3 The induction of PMR4 in response to OGs is reduced in pcap1‐mutant plants. [file PCE-44-3078-s005.pdf]

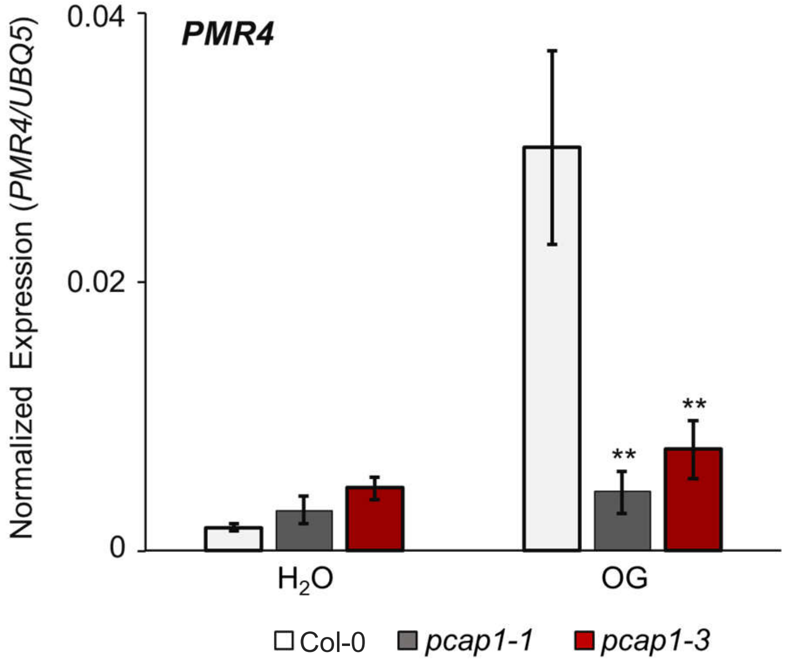

Figure S3. The induction of *PMR4* in response to OGs is reduced in *pcap1* mutant plants. *PMR4* expression analysis in water- and OG-infiltrated Col-0 and *pcap1* null mutant leaves after 24 hours infiltration. *PMR4* expression was analyzed 24 h after infiltration by qRT-PCR using *UBQ5* as reference. Bars indicate average expression of three independent biological replicates. Asterisks indicate statistically significant differences between mutant and wild-type lines according to Student's t test (*, $P < 0.05$; **, $P < 0.01$; ***, $P < 0.001$).
