# Supplementary material for: The plasma membrane–associated Ca2+ ‐binding protein, PCaP1, is required for oligogalacturonide and flagellin‐induced priming and immunity
Source: Plant Cell Environ. 2021 Jun 30;44(9):3078–93. doi: 10.1111/pce.14118 (PMC8457133; doi:10.1111/pce.14118)
Supplement: Supplementary file 5 — Figure S5 Quantitative imaging analysis of OG‐ and flg22‐induced endocytosis in proPCaP1:PCaP1‐GFP seedlings. [file PCE-44-3078-s004.pdf]

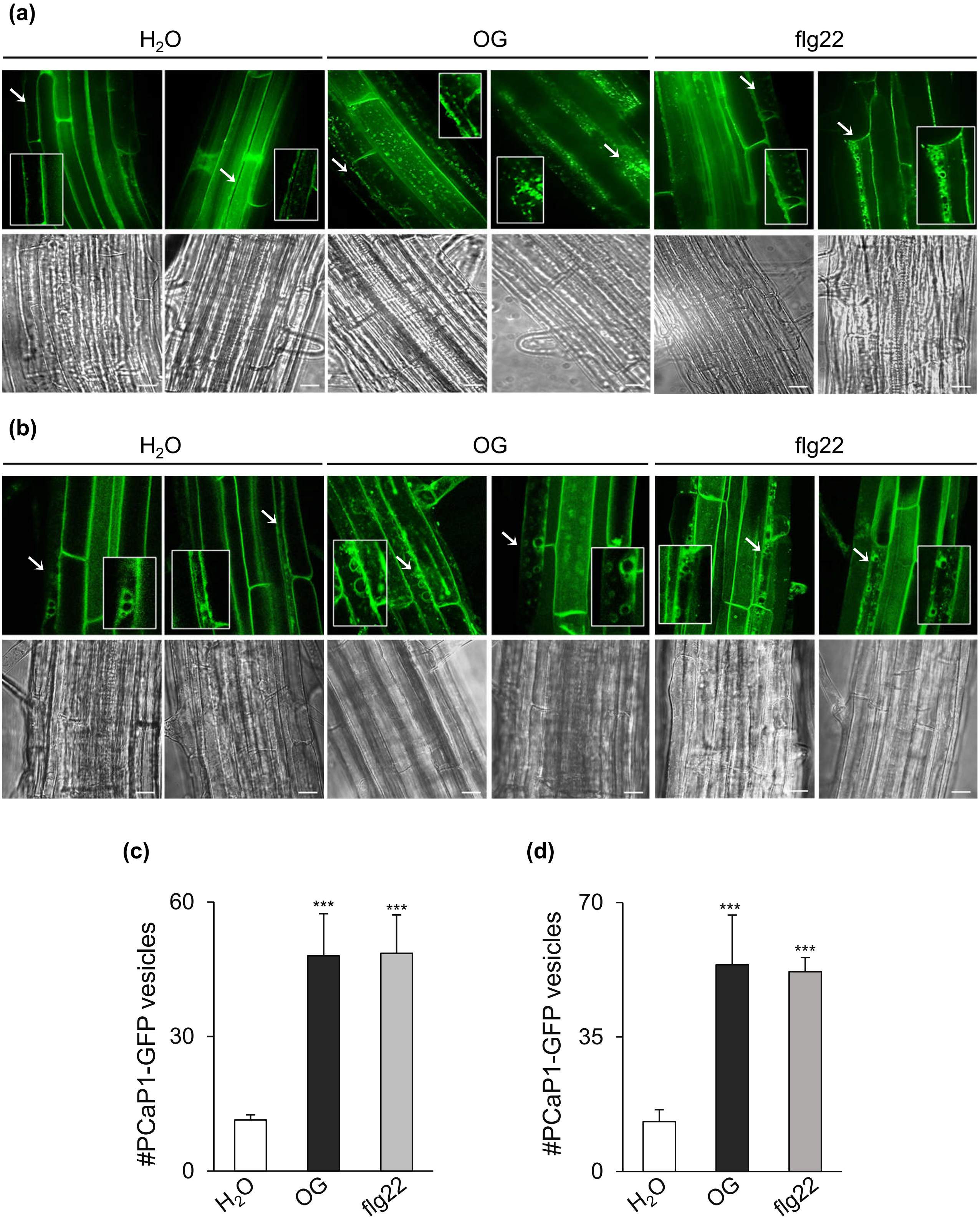

Figure S5. Quantitative imaging analysis of OG- and flg22-induced endocytosis in proPCaP1:PCaP1-GFP seedlings. Cell cortex optical sections of epidermal root cells in H2O-, OG- and flg22-treated proPCaP1-GFP seedlings by confocal spinning disk (a) and confocal laser scanning (b) microscopy (two images for each treatment); the corresponding bright-field microscopy images are shown. Insets: magnification of areas of interest. Bars = 20 µm. (c and d) Quantification of the PCaP1-GFP-positive vesicle in the images shown in (a) and (b), respectively, expressed as number of vesicles per image area.
