# Supplementary material for: The plasma membrane–associated Ca2+ ‐binding protein, PCaP1, is required for oligogalacturonide and flagellin‐induced priming and immunity
Source: Plant Cell Environ. 2021 Jun 30;44(9):3078–93. doi: 10.1111/pce.14118 (PMC8457133; doi:10.1111/pce.14118)
Supplement: Supplementary file 6 — Figure S6 Confocal microscopy micrographs of root epidermal cells in response to OGs in null mutants. [file PCE-44-3078-s001.pdf]

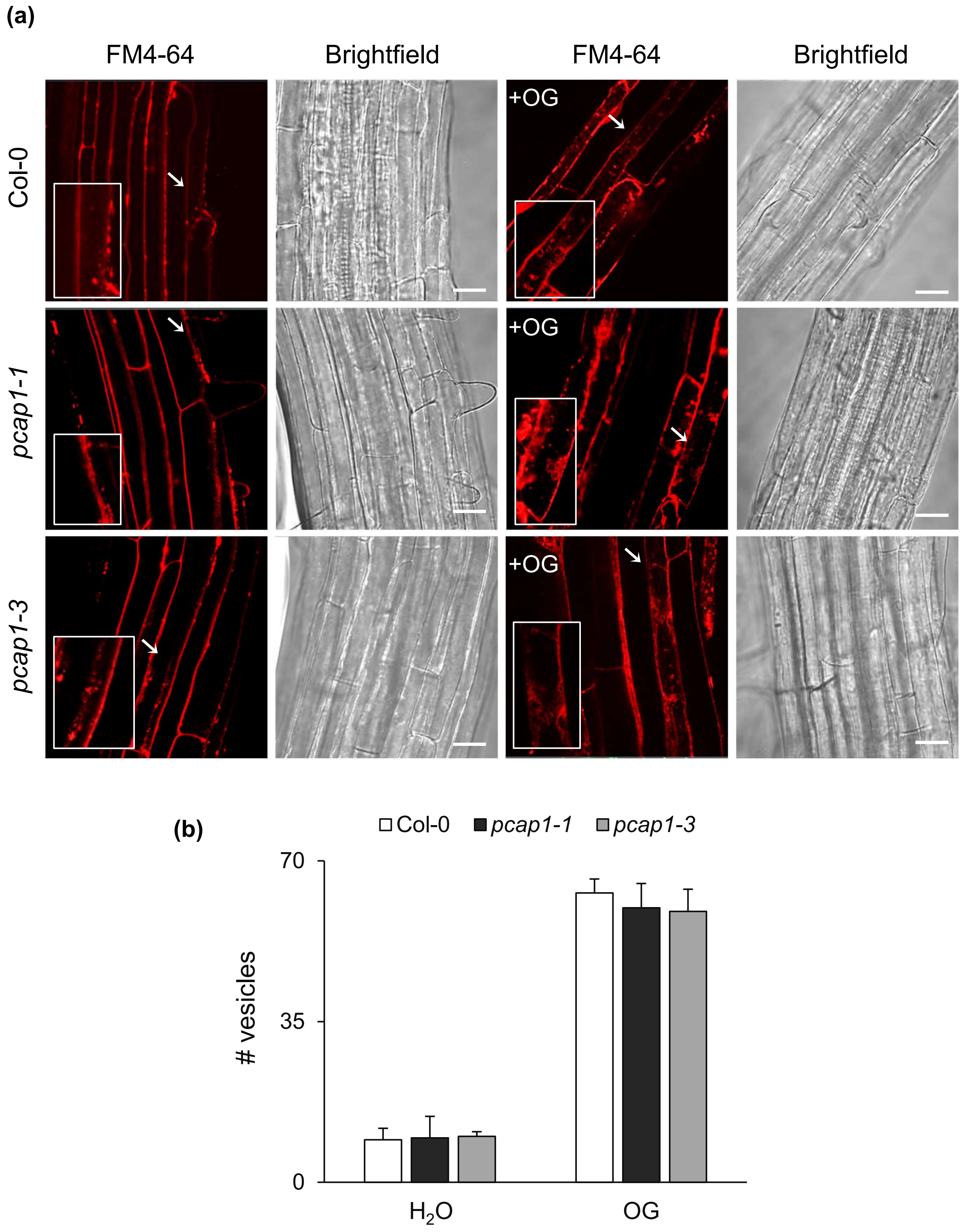

Figure S6. Confocal microscopy micrographs of root epidermal cells in response to OGs in null mutants. (a) Roots of wild type and *pcap1* null mutant seedlings stained with endocytic tracer FM4-64 (2 μM) after OG treatment [FM4-64 labelling: red]. The corresponding bright-field microscopy images are shown. (b) Quantification of FM4-64-positive vesicle in the images shown in (a) expressed as number of vesicles per image area in the absence and presence of treatment. Insets: magnification of areas of interest. Bars = 20 μm.
