# Supplementary material for: The plasma membrane–associated Ca2+ ‐binding protein, PCaP1, is required for oligogalacturonide and flagellin‐induced priming and immunity
Source: Plant Cell Environ. 2021 Jun 30;44(9):3078–93. doi: 10.1111/pce.14118 (PMC8457133; doi:10.1111/pce.14118)
Supplement: Supplementary file 7 — Table S1. Primers used in this work. [file PCE-44-3078-s006.docx]

**Table S1.** Primers used in this work.

| **GENE** | **AGI CODE** | **FORWARD PRIMER (5’-3’)** | **REVERSE PRIMER (5’-3’)** |
| --- | --- | --- | --- |
| ***PCaP1*** | **AT4G20260** | GGTGTTTATCTACGGGTTGGAG | CTCCGGGAGGAAAACAGATAC |
| ***PCaP1*** | **AT4G20260** | ATGGGTTACTGGAATTCCAA | CTCCGGGAGGAAAACAGATAC |
| ***PCaP1*** | **AT4G20260** | GGTGAGTGAGGCTTCGTCTA | TCACCACTTGTTCCTTCGGT |
| ***UBQ5*** | **AT3G62250** | GTTAAGCTCGCTGTTCTTCAGT | TCAAGCTTCAACTCCTTCTTTC |
| ***RET-OX*** | **AT1G26380** | AGGTTCTCGAACCCTAACAACA | GCACAGACGACACGTAAGAAAG |
| ***FRK1*** | **AT2G19190** | TTAAACTCGACGATGCAACA | GATGGAAGTTTTCCCGTTTT |
| ***CYP81F2*** | **AT5G57220** | GTGAAAGCACTAGGCGAAGC | ATCCGTTCCAGCTAGCATCA |
| ***PAD3*** | **AT3G26830** | CCGGTGAATCTTGAGAGAGCC | GATCAGCTCGGTCATTCCCC |
| ***PGIP1*** | **AT5G06860** | TCTTGAACTTAGCAGGAAC | GAGAGCTGGTTATGTGATAG |
| ***PDF1.2*** | **AT5G44420** | CGCACCGGCAATGGTGG | ATCCATGTTTGGCTCCTTCG |
| ***PR1*** | **AT2G14610** | GTAGGTGCTCTTGTTCTTCCC | CACATAATTCCCACGAGGATC |
| ***PMR4*** | **AT4G03550** | AGTTCAAGGACGGCATTCAT | AAGCTCATTGCACAACAAGA |

Sequence primers was designed by using Primer3web (http://bioinfo.ut.ee/primer3/). Primers used for genotyping are underlined.
